# Enhanced MobileNet with multi-scale feature fusion for automated breast cancer histopathology classification

**DOI:** 10.1038/s41598-026-60967-z

**Published:** 2026-07-15

**Authors:** Mohamed E. Ali, Atef Z. Ghalwash, Amany Abdo

**Affiliations:** 1 Department of Business Information Systems, Higher Institute of Computers and Information Technology, Elshorouk Academy, Cairo, Egypt; 2 Department of Computer science, Faculty of Computers and Artificial Intelligence, Helwan University, Cairo, Egypt; 3 Department of Information Systems, Faculty of Informatics and Computer Science, The British University, Cairo, Egypt; 4 Department of Information Systems, Faculty of Computers and Artificial Intelligence, Helwan University, Cairo, Egypt

**Keywords:** Convolutional neural networks, Breast cancer detection, Transfer learning, Deep learning, Histopathology, Multi-scale feature fusion, Medical image analysis, Cancer, Computational biology and bioinformatics, Mathematics and computing, Oncology

## Abstract

Accurate and efficient diagnosis of breast cancer from histopathological images remains a major challenge in clinical practice due to subjective interpretation, inter-observer variability, and labor-intensive manual examination. To address these limitations, this work introduces a transfer learning–based framework for automated breast cancer classification using the Breast Cancer Histology Images (BACH) dataset. Several pre-trained deep architectures—including MobileNet, ResNet variants, EfficientNet, and Vision Transformers—were evaluated and extended with a Multi-Scale Feature Fusion (MSFF) module to capture morphological heterogeneity across spatial resolutions. Among these, the Enhanced MobileNet (E‑MobileNet) with MSFF outperforming recent state‑of‑the‑art models and achieving a classification accuracy of 95%, precision of 95%, recall of 94%, and F1‑score of 96%. The framework was further validated on the BreaKHis dataset across multiple magnifications, achieving an average accuracy of 90.6%. These results confirm the robustness and generalization capability of the proposed model for practical clinical deployment in digital pathology.

## Introduction

**Fig. 1:**
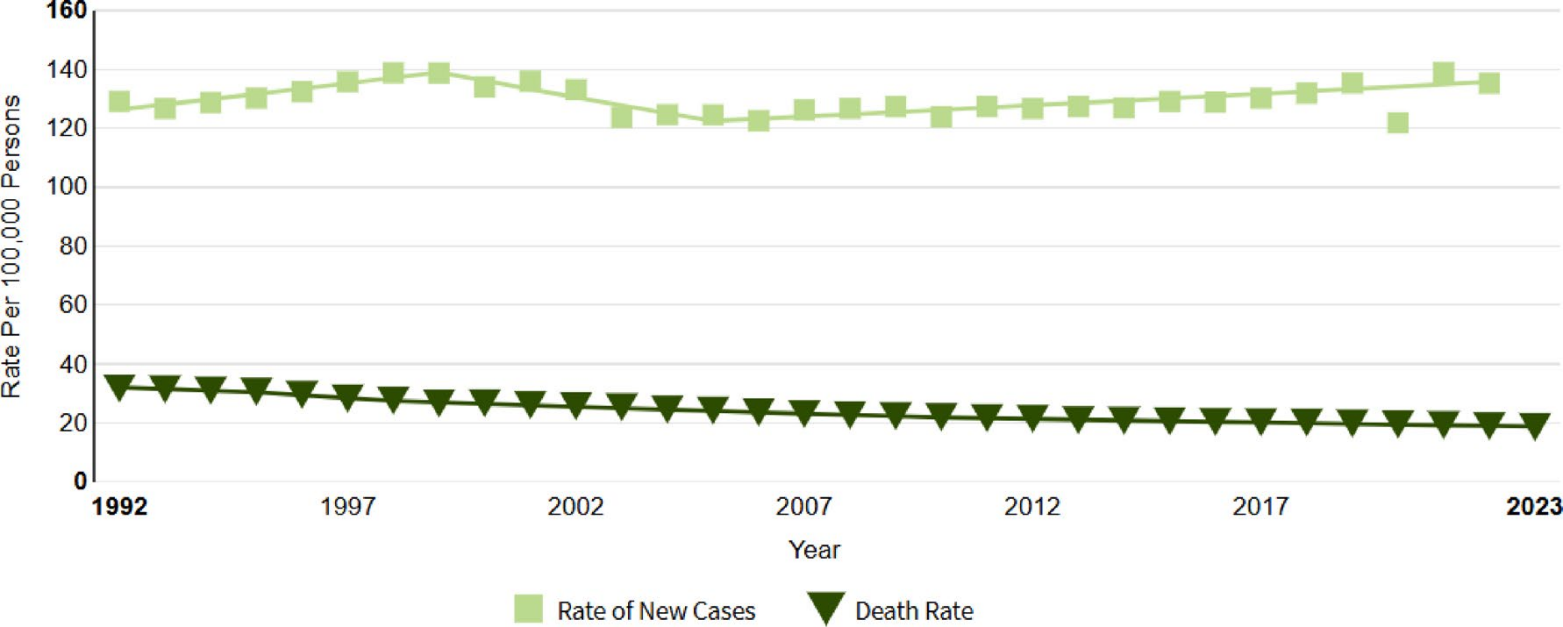
Cancer statistics facts: female breast cancer^2^.

Breast cancer remains one of the most significant health challenges facing women globally, with approximately 13.0% of women expected to be diagnosed with the disease during their lifetime. The disease affects over 4 million women currently living with breast cancer in the United States alone, with an annual incidence rate of 130.8 per 100,000 women and a death rate of 19.2 per 100,000 women per year. Despite advances in treatment, breast cancer continues to be the fourth leading cause of cancer death in the United States, with survival outcomes heavily dependent on early detection and intervention as shown in Fig. 1. The critical importance of early detection is underscored by the substantially higher survival rates achieved when breast cancer is identified and treated during its initial stages, making the development of more accurate and efficient detection methods a paramount healthcare priority^1^.

The standard breast cancer diagnostic workflow follows a well-established multi-step pathway. It begins with clinical breast examination and patient history assessment, followed by imaging evaluation using mammography, ultrasound, or magnetic resonance imaging (MRI) to identify suspicious lesions. Suspicious findings are subsequently confirmed through histopathological examination of biopsied tissue, which remains the gold standard for definitive diagnosis. In addition, immunohistochemical (IHC) staining is routinely performed to characterize molecular subtypes (e.g., estrogen receptor, progesterone receptor, and HER2/neu status), which guides treatment decisions and prognosis^2^. This multi-modal diagnostic pathway highlights the complexity of breast cancer diagnosis and underscores the need for AI-assisted tools that can enhance accuracy, reduce inter-observer variability, and accelerate diagnostic workflows across different stages of this pipeline.

Traditional mammographic screening has served as the cornerstone of breast cancer detection since the 1970 s, fundamentally transforming early detection capabilities by identifying malignancies before clinical symptoms manifest. Current screening guidelines recommend annual mammograms beginning at age 40 for average-risk women, with more frequent screening for those at higher risk. While mammography has demonstrated significant success in reducing breast cancer mortality and saving countless lives, technology faces inherent limitations that impact its diagnostic accuracy. Breast cancer remains one of the most significant health concerns among women worldwide, and continuous technological innovation has greatly improved early diagnostic capabilities. Digital breast tomosynthesis (DBT), commonly known as 3D mammography, represents a major advancement in breast imaging, enabling layered visualization of breast tissue that assists in detecting minute calcifications, microlesions, and other early cancer indicators. Similarly, molecular breast imaging (MBI) offers additional diagnostic value, particularly for women with dense breast tissue or those with moderate cancer risk, by employing targeted radioactive tracers to identify regions of elevated metabolic activity that may correspond to malignant growth^1^. Despite these technological improvements, traditional screening methods continue to rely heavily on expert radiologists, whose interpretations may vary due to fatigue, subjective bias, or the complexity of dense breast structures^3^.

The recent integration of artificial intelligence (AI) into diagnostic imaging has introduced a transformative shift in how breast cancer is detected, analyzed, and managed. AI techniques are now widely applied in four main domains: image interpretation, workflow optimization, predictive and personalized diagnostics, and clinical decision support^4^. Advanced deep learning algorithms have demonstrated the capacity to identify subtle structural anomalies that are sometimes overlooked by human observers, while also minimizing inter-observer variability and improving diagnostic efficiency^5^. Several comparative studies have shown that AI-based systems achieve performance comparable to that of expert radiologists in mammographic screening, and in some cases even surpass them in specificity while maintaining high sensitivity. Moreover, combining AI algorithms with human evaluation has been shown to achieve diagnostic accuracy similar to the double-reading standard while substantially improving speed and consistency. Beyond accuracy gains, AI-driven automation contributes to reduced healthcare costs and greater operational efficiency in breast cancer screening^6^.

In recent years, multimodal approaches integrating imaging data with clinical, genomic, and pathological information have gained significant attention in breast cancer research. For instance, studies have demonstrated that combining histopathological images with genomic biomarkers can improve prognostic accuracy and enable more personalized treatment strategies^7^. Similarly, the integration of radiological imaging (mammography, ultrasound, MRI) with histopathology and IHC data has shown promise in providing a more comprehensive understanding of tumor heterogeneity and behavior^8^. These developments highlight the potential of AI to facilitate multimodal breast cancer diagnosis and contribute to precision oncology, where treatment decisions are tailored to individual patient characteristics^9, 10, 11^.

Transfer learning has emerged as a pivotal technique in medical imaging AI, particularly for tasks where large, annotated datasets are difficult to obtain. It allows deep neural networks pre-trained on large-scale image databases to be adapted for specialized domains such as mammography or histopathology^12^. By leveraging features learned from millions of general images, transfer learning accelerates convergence, reduces computational cost, and frequently achieves higher performance than models trained from scratch. This is especially valuable for breast cancer diagnosis, where accurate classification depends on recognizing subtle visual patterns across diverse tissue morphologies and densities^13^.

Recent advances in computational pathology have increasingly focused on multi-scale feature extraction to capture the hierarchical nature of histopathological images. Shallow layers of convolutional neural networks (CNNs) capture fine-grained details such as cell boundaries and nuclear textures, while deeper layers encode high-level semantic information such as tissue architecture and lesion morphology. Multi-Scale Feature Fusion (MSFF) techniques aim to combine these complementary representations to improve classification performance^14^. While several fusion strategies have been proposed—including Feature Pyramid Networks (FPN)^15^, Path Aggregation Networks (PANet)^16^, and Bi-directional Feature Pyramid Networks (BiFPN)^17^—most of these approaches were designed for natural image object detection and may not optimally address the unique challenges of histopathology, where both fine-grained cellular details and broader tissue-level context are equally critical.

This paper proposes an Enhanced MobileNet architecture with a novel Multi-Scale Feature Fusion (MSFF) module tailored specifically for breast cancer histopathology classification. Unlike existing fusion approaches that primarily focus on object detection in natural images, our MSFF module is designed to address three critical challenges in computational pathology: (1) limited dataset size, (2) stain variability in histopathology images, and (3) the need for clinically interpretable results. The proposed MSFF module differs from existing strategies in three key aspects: (a) it fuses features from carefully selected intermediate layers of MobileNetV3-Large that correspond to biologically meaningful scales (cellular, nuclear, and tissue levels), (b) it employs adaptive pooling and channel-wise attention to weight the contribution of each scale dynamically, and (c) it achieves superior performance on the BACH dataset while maintaining computational efficiency suitable for deployment in resource-constrained clinical settings. MobileNet was selected as the backbone architecture due to its exceptional balance between accuracy and computational efficiency, making it ideal for deployment-oriented applications on standard hardware, unlike heavier architectures such as ResNet-152 or Vision Transformers that require substantial computational resources.

The contributions of this work are summarized as follows:

- It introduces a novel Multi-Scale Feature Fusion (MSFF) module integrated with the MobileNetV3-Large backbone, achieving 95% accuracy on the BACH dataset.
- The proposed method was designed to address three critical challenges in computational pathology: (1) limited dataset size, (2) stain variability in histopathology images, and (3) the need for clinically interpretable results.
- The workflow systematically progresses through data preparation, model development, and deployment phases.

The rest of paper is organized as follows: Sect. 2 discusses related work; Sect. 3 describes the proposed method; Sect. 4 presents the experimental results and discussion; Sect. 5 Comparison with state-of-the-art methods, and Sect. 5 concludes the study and outlines future research directions.

.

## Related works

Breast cancer remains a leading cause of mortality among women worldwide, underscoring the necessity for accurate and timely diagnostic solutions. Recent research has explored the use of artificial intelligence to improve detection accuracy, particularly through deep transfer learning for classification and U-Net architectures for segmentation. In study^6^, a cost-effective and accessible web-based Breast Cancer Prediction System (BCPS) was proposed to support early risk identification. This system classifies women into three risk categories—low, moderate, and high—based on genetic and environmental factors. It utilizes data mining techniques and the WEKA analytical platform to process patient data and evaluate algorithmic performance. Among the compared algorithms, C4.5 achieved the best results with 95.61% accuracy and the lowest error rate of 4.38%, outperforming RandomTree, REPTree, and SimpleCart classifiers. The BCPS provides an interactive and accessible diagnostic aid that promotes early screening and prevention at minimal cost and effort.

Transfer learning continues to be a dominant technique in medical imaging, primarily due to the scarcity of large, annotated datasets. In^16^ utilized ultrasound breast images categorized into normal, benign, and malignant classes to evaluate deep learning models. The authors implemented pre-trained CNN architectures—VGG16, VGG19, and EfficientNet—through transfer learning to extract discriminative visual patterns, while employing a U-Net network for precise tumor boundary segmentation. Among these models, VGG19 achieved the best classification performance in terms of accuracy, precision, and recall, whereas U-Net produced an average Dice Similarity Coefficient of 85.97%, confirming its effectiveness in delineating lesions. Together, these models formed an integrated diagnostic framework capable of improving lesion localization, reducing observer variability, and enhancing clinical decision support. Although the approach demonstrated strong diagnostic potential, challenges such as computational overhead, limited dataset diversity, and unbalanced class distributions were noted as barriers to broader clinical adoption. In^17^ compared CNN models trained from scratch with those fine-tuned via transfer learning across several medical imaging domains and found consistent performance gains for the latter approach. Similarly^18^, demonstrated the effectiveness of transfer learning for histopathological image analysis, showing that adapting ImageNet-trained models significantly improved classification accuracy compared to training specialized models from the ground up.

Further, a recent study^19^ proposed a hybrid diagnostic framework that combines deep learning with handcrafted features for classifying breast lesions in ultrasound scans. The authors experimented with multiple CNN backbones—InceptionV3, EfficientNetB4, ResNet50, and VGG16—and integrated them with manually extracted texture features using support vector machines. A late-fusion strategy was applied to merge their predictions, resulting in improved diagnostic accuracy. The ResNet50 model achieved an F1-score of 81.97%, while the ensemble configuration increased this to 83.90%. When validated across independent datasets, the ensemble achieved F1-scores of 88.70% and 78.20% on the BUSI and BUID datasets, respectively. These findings emphasize that combining pre-trained networks with classical image features enhances model robustness and adaptability across different imaging datasets.

In^20^ addressed the problem of class imbalance in histopathological image classification by employing VGG19 as a base architecture under a transfer learning framework with advanced data balancing and augmentation techniques. This approach led to a noticeable performance improvement, demonstrating the critical role of data balancing in medical image analysis. However, since the method was trained exclusively on histopathology data, its applicability to other imaging modalities like ultrasound and mammography remains uncertain.

In^21^ shifted focus towards predictive modeling, proposing a deep learning framework for breast cancer risk prediction using mammography data. By leveraging CNN architectures, the model successfully predicted the likelihood of breast cancer occurrence within a 1–5 year horizon. This contribution is particularly valuable for preventive healthcare, as it emphasizes early intervention and patient monitoring. However, the model required longitudinal patient data, which is often difficult to acquire, thus potentially limiting large-scale deployment in diverse healthcare settings.

In^22^ presented a hybrid machine learning approach aimed at both detection and prevention of breast cancer. The study combined multiple machine learning algorithms into an ensemble framework to improve robustness and predictive power. The system also incorporated treatment recommendation capabilities, offering a more holistic clinical decision-support tool. Despite its promising results in early detection and treatment suggestions, the framework faced challenges related to computational complexity which are critical for widespread accessibility and scalability, Table 1 provides a summary of pertinent earlier research on breast cancer detection models.

**Table 1:** Summarization of breast cancer detection model.

Study (Ref, Title, Year)	Methodology/Techniques	Performance Metrics	Limitations/gaps
^6^ Synergistic transfer learning and adversarial networks for breast cancer diagnosis (2025)	Deep Learning with GANs and Transfer Learning – Conditional Wasserstein GAN (cWGAN) for data augmentation and CNN with transfer learning	Improved classification accuracy through synthetic data generation	Limited to benign vs. invasive classification; high computational cost
^17^ Deep Learning in Breast Cancer Imaging: State of the Art and Recent Advancements (2025)	Comprehensive review of CNN, ResNet, VGG, DenseNet architectures for mammography, ultrasound, and MRI	Qualitative synthesis of diagnostic precision and workflow efficiency	Review paper; no specific performance metrics
^18^ Machine Learning and New Insights for Breast Cancer Diagnosis (2024)	AI-based Computer-Aided Diagnosis (CAD) using U-Net for segmentation and thermography-based analysis	89.03% intersection-over-union (IoU)	Limited to thermography imaging; requires specialized equipment
^19^ Machine-Learning Methods in Detecting Breast Cancer and Related Therapeutic Issues (2024)	Review of ML algorithms (SVM, Random Forest, Neural Networks) for tumor size analysis and early detection		
^20^ Classification of Breast Cancer Using Transfer Learning and Advanced Al-Biruni Earth Radius Optimization (2024)	Transfer Learning with pre-trained CNNs (VGG, ResNet) + optimization algorithm	Enhanced feature extraction and accuracy	High algorithmic complexity; limited dataset validation
^23^ Imbalanced Breast Cancer Classification Using Transfer Learning (2020)	VGG-19 base model with data augmentation and balancing techniques	Improved classification on imbalanced datasets	Focused on histopathology only; limited modality generalization
^21^ Deep Learning Model for Breast Cancer Risk Prediction (2021)	CNN models for 1–5 year mammography-based risk prediction	Accurate long-term risk forecasting	Requires longitudinal data; limited scalability
^22^ Breast Cancer Detection and Prevention Using Machine Learning (2024)	Hybrid ML ensemble for detection and treatment recommendation	Early detection and treatment guidance	High computational complexity

## Methodology

### The proposed model

**Fig. 2:**
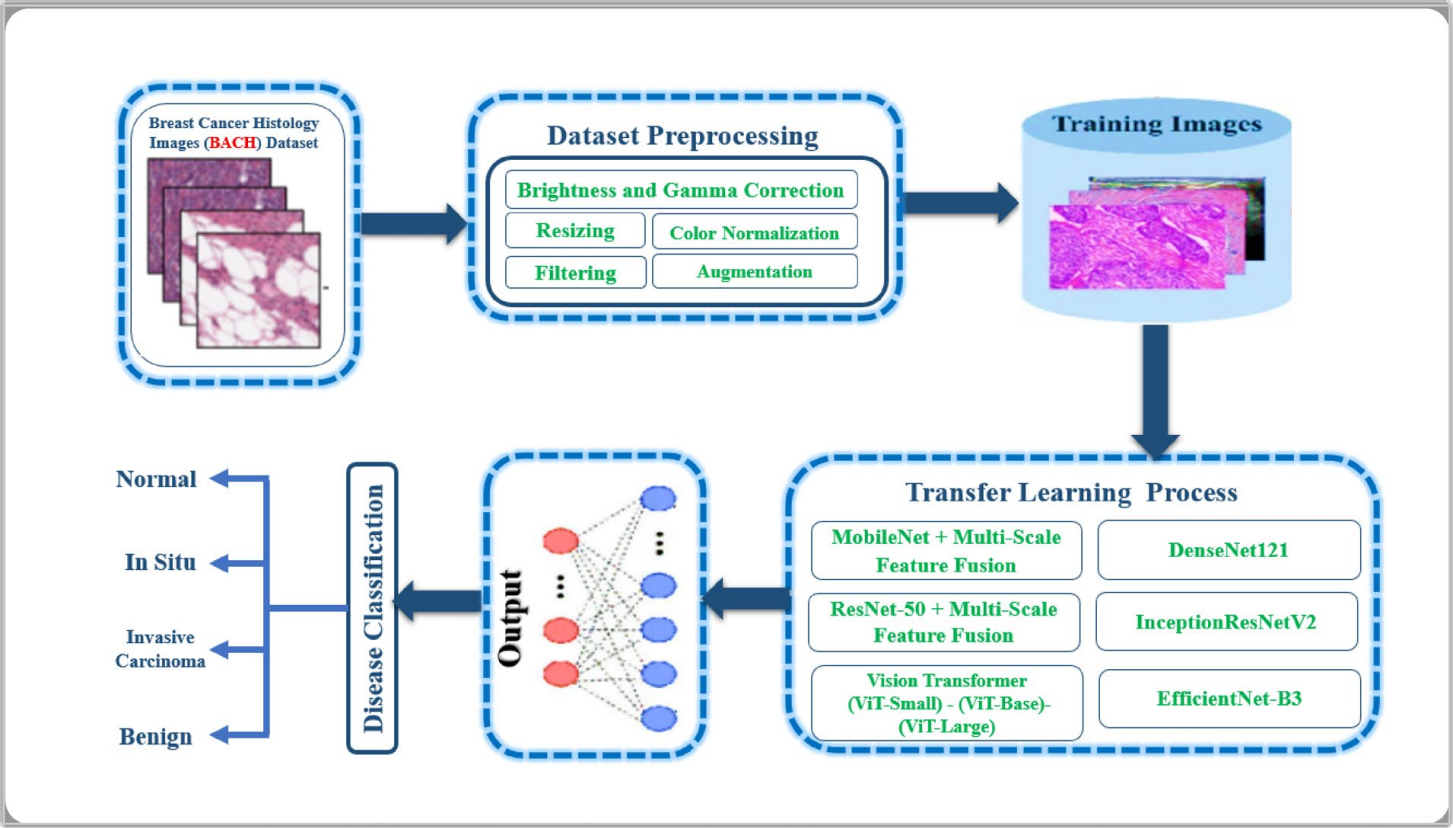
Transfer learning architecture with multi-scale feature fusion for breast cancer histology image classification.

This section presents the proposed model for breast cancer detection. As illustrated in Fig. 2, the workflow for classifying breast cancer histology images from the BACH dataset. It consists of three main phases: data collection, data preprocessing, and disease classification through a transfer learning-based deep learning pipeline. It starts with dataset preprocessing, including brightness and gamma correction, resizing, color normalization, filtering, and augmentation to enhance image quality. The preprocessed images are used as training input for various transfer learning models, such as MobileNet, ResNet-50, Vision Transformer, DenseNet121, InceptionResNetV2, and EfficientNet-B3, often combined with multi-scale feature fusion. These models extract features and generate outputs to classify the images into four categories: normal, in situ, invasive carcinoma, and benign.

In the data collection phase, we utilized the BACH dataset, which contains microscopic tissue samples used for histopathological analysis. Following preprocessing, the images are processed within a transfer learning model that integrates multiple state-of-the-art deep neural network architectures. These include MobileNet with multi-scale feature fusion for efficient deployment, ResNet-50 with multi-scale feature fusion to exploit residual connections, Vision Transformer (ViT) variants (ViT-Small, ViT-Base, and ViT-Large) leveraging attention mechanisms, DenseNet121 for improved feature propagation, InceptionResNetV2 combining inception modules with residual learning, and EfficientNet-B3 for optimized scaling. The multi-scale feature fusion component plays a key role in combining features extracted at different resolutions, enabling the model to capture both fine-grained cellular details and broader tissue-level patterns.

Finally, in the classification phase, the extracted and fused features are passed through the disease classification module, which categorizes histological images into four clinically relevant classes: Normal tissue, In Situ carcinoma, Invasive carcinoma, and Benign. By leveraging transfer learning and multi-architecture fusion, the proposed model addresses the limitations of small medical datasets and provides a robust, accurate, and efficient tool to assist pathologists in breast cancer diagnosis from histopathological images.

### Datasets

####  Breast cancer histology image (BACH)

**Fig. 3:**
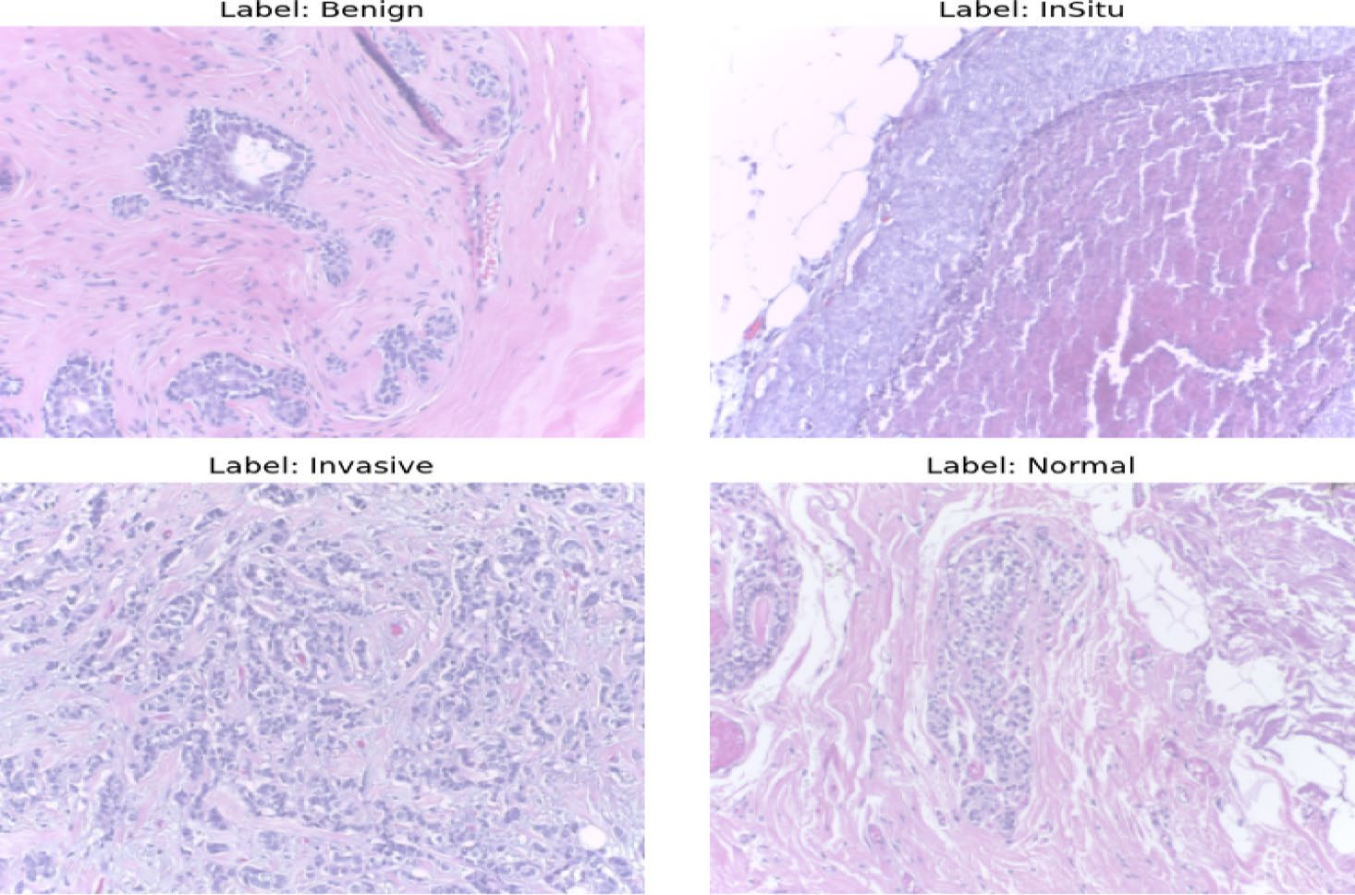
Samples of histology image of benign breast tissue of the BACH dataset.

In^24^, BACH (Breast Cancer Histology) dataset which comprises 400 high-resolution (2048 × 1536 pixel) histopathology images evenly distributed across four diagnostic categories: normal tissue exhibiting regular glandular architecture, benign lesions demonstrating non-cancerous abnormalities, in situ carcinoma (DCIS) showing confined malignant proliferation, and invasive ductal carcinoma featuring stromal invasion patterns. Ground-truth labels are distributed via CSV. These images define Part A of the challenge (4-class image-wise classification). Figure 3 presents representative samples from each of these four classes, arranged in a grid to illustrate their distinct histopathological characteristics. The normal tissue shows organized glandular structures with uniform nuclei, the benign lesions maintain intact basement membranes with mild structural variations, the insitu carcinoma demonstrates localized malignant cell proliferation within ducts, and the invasive carcinoma exhibits disrupted tissue organization and stromal invasion.

**Fig. 4:**
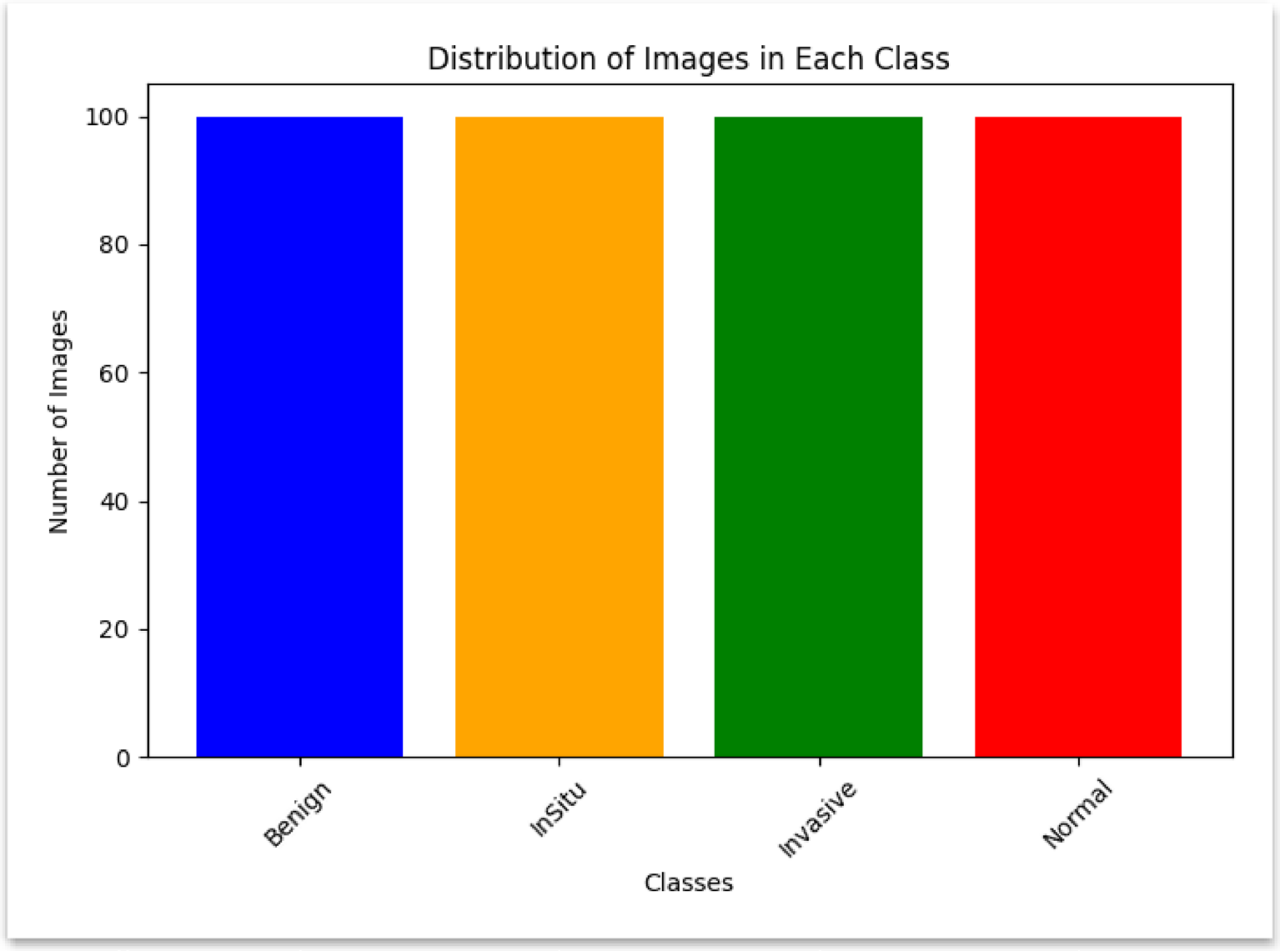
Distribution of images across breast cancer histology classes.

The dataset’s balanced class distribution is demonstrated in Fig. 4, where each diagnostic category contains precisely 100 samples. This equitable distribution prevents model bias during training while reflecting real-world clinical prevalence ratios according to recent epidemiological studies^25^. The benign samples particularly showcase a spectrum of fibrocystic changes and adenosis patterns that provide critical negative examples for teaching the model to distinguish between truly malignant and benign-but-morphologically-complex tissue architecture.

#### Breast cancer histopathological database (BreaKHis)

**Fig. 5:**
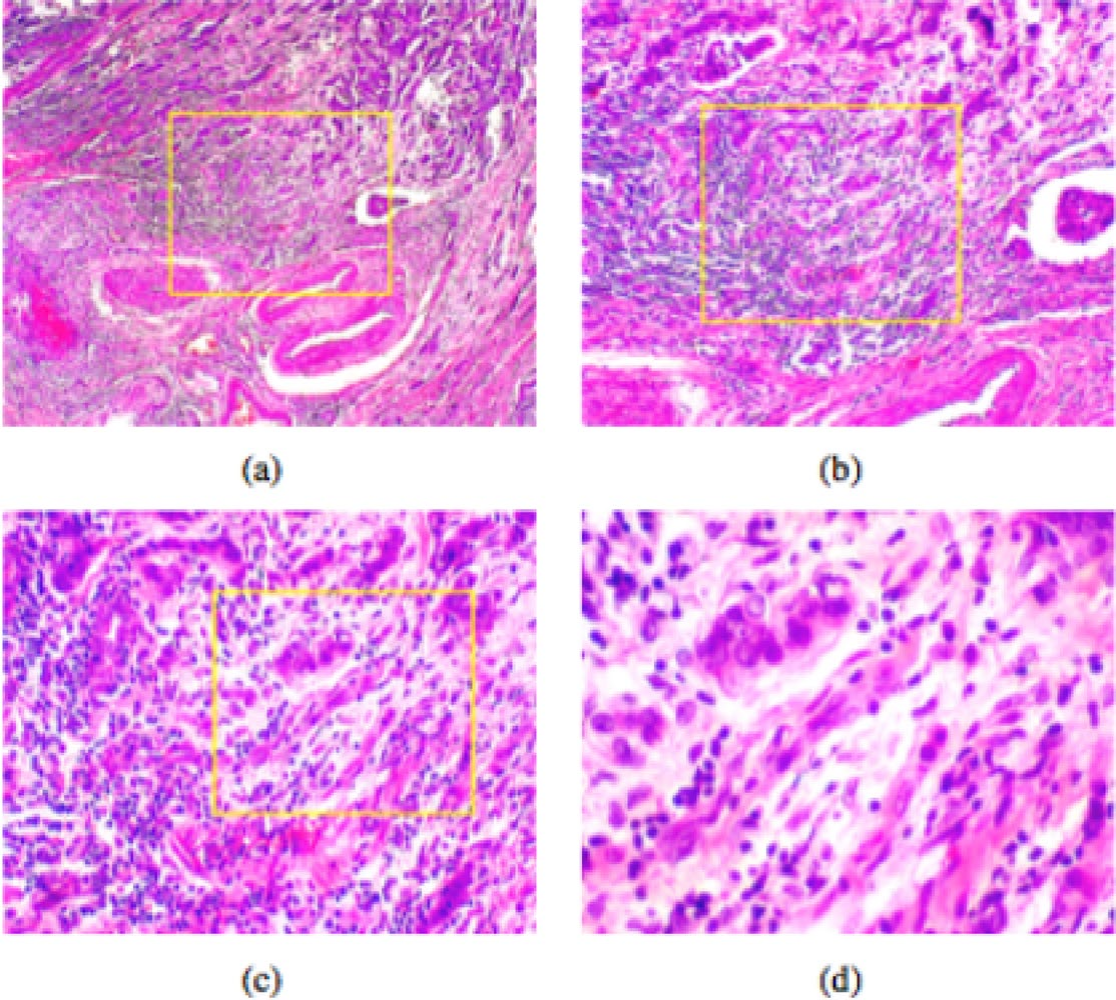
A slide of breast malignant tumor (stained with HE) seen in different magnification factors: (**a**) 40X, (**b**) 100X, (**c**) 200X, and (**d**) 400X.

In^26^, BreaKHis is a public H&E breast tumor dataset containing 9,109 RGB PNG microscopy images from 82 patients across four magnifications (40×, 100×, 200×, 400×) as shown in Fig. 5. Images are 700 × 460 px, 8-bit per channel, and labeled as benign (3,680) or malignant (5,429) with eight subtypes (benign: adenosis, fibroadenoma, phyllodes tumor, tubular adenoma; malignant: ductal, lobular, mucinous, papillary). Filenames encode biopsy method (SOB/excisional), class, subtype, year, slide ID, magnification, and sequence, enabling reproducible splits.

### Data preprocessing

In data preprocessing phase, several operations were applied to enhance image quality and prepare the data for model training. These include brightness and gamma correction to normalize lighting conditions, resizing to ensure uniform input dimensions, color normalization to reduce staining variations, filtering to remove artifacts, and data augmentation to increase the diversity and size of the training dataset.

The experimental methodology was developed through systematic evaluation of preprocessing techniques applied to the BACH dataset^27^, which was partitioned using stratified sampling to maintain balanced class distributions. The dataset was divided into training (80%, 320 images) and testing (20%, 80 images) sets, ensuring proportional representation of all four diagnostic categories in both subsets. This partitioning strategy preserved the original class distribution while preventing data leakage between sets.

Initial preprocessing addressed illumination variability through gamma correction, implemented as:

1 $$\:{I}_{corrected}\left(x,y\right)=255\times\:{\left(\frac{{I}_{original}\left(x,y\right)}{255}\right)}^{{\upgamma\:}}$$

with γ ∈ [0.7,1.3] optimized to enhance nuclear visibility in overexposed regions while preserving cytoplasmic detail^28^. Spatial standardization was achieved through bilinear interpolation resizing to 224 × 224 pixels, a dimension selected for compatibility with standard deep learning architectures^29^.

Multiple color normalization approaches were rigorously evaluated. Reinhard normalization transformed images into CIELAB color space and aligned channel statistics to a reference image. Macenko normalization^29^ operated in optical density space through singular value decomposition^30^, while conventional min-max and per-channel RGB normalization served as baseline comparisons. The selected ImageNet normalization scheme:

2 $$\:{I}_{norm}=\frac{I-\left[\mathrm{0.485,0.456,0.406}\right]}{\left[\mathrm{0.229,0.224,0.225}\right]}$$

demonstrated superior compatibility with pretrained networks while maintaining diagnostic validity as confirmed by pathologist review.

The augmentation strategy incorporated geometric and photometric transformations implemented through PyTorch’s torchvision.transforms. Random horizontal and vertical flips (*p* = 0.5) and constrained rotations (± 20°) preserved tissue orientation cues, while color jittering in HSV space introduced biologically plausible variations. These transformations were carefully limited to maintain histopathological validity, with parameter ranges determined through iterative validation with clinical experts during the original collection of the data. All augmentation was applied on the fly during training, meaning that the same 400 original images were dynamically transformed across different epochs rather than permanently expanded. This approach effectively increased the diversity of training samples while maintaining the original dataset size.

**Fig. 6:**
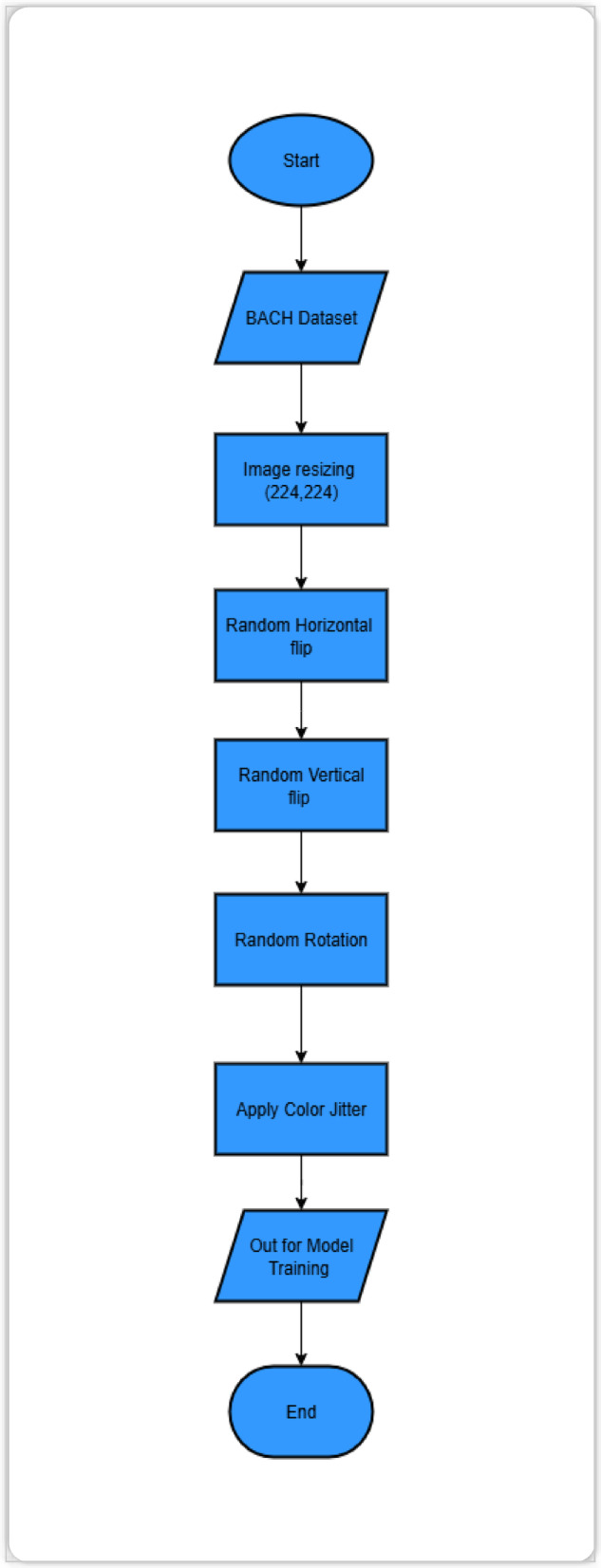
Preprocessing and data augmentation flowchart for histopathological images.

Two regularization techniques were integrated into the training pipeline. Label smoothing^31^ modified the target distribution to prevent model overconfidence:

3 $$\:{q}_{i}^{{\prime\:}}=\left(1-\epsilon\right){q}_{i}+\frac{\epsilon}{K}$$

with ε = 0.1 and K = 4 classes. Mixup augmentation^31^ generated synthetic training examples through linear interpolation of both images and labels^32^:

4 $$\:{I}_{mix}={\uplambda\:}{I}_{i}+\left(1-{\uplambda\:}\right){I}_{j}\:$$

5 $$\:{y}_{mix}={\uplambda\:}{y}_{i}+\left(1-{\uplambda\:}\right){y}_{j}$$

where λ ∼ Beta(α,α) with α = 0.4. The complete preprocessing pipeline was implemented on Kaggle’s GPU-accelerated platform (NVIDIA Tesla P100) with fixed random seeds (seed = 42) to ensure reproducibility across experiments. The representation of the pipeline is shown in Fig. 6.

### Training strategy

A hold-out validation approach (80/20 split) was employed to ensure consistent evaluation. The model was trained for 10 epochs, empirically determined to achieve convergence while minimizing overfitting. The final training and validation accuracies reached 98.4% and 95%, respectively, indicating minor overfitting within an acceptable margin for limited medical imaging datasets. CrossEntropyLoss served as the primary objective function due to its effectiveness in multi-class classification tasks, while optimization was performed using the Adam algorithm with model-specific learning rates ranging from 1e-4 to 1e-5 based on architectural complexity. Learning rate adaptation was implemented through ReduceLROnPlateau schedulers configured with mode=’max’, factor = 0.5, and patience = 2 epochs to monitor validation accuracy plateaus.

Model performance was rigorously assessed using a comprehensive suite of metrics including accuracy, macro-average F1-score, per-class precision and recall, and multi-class ROC-AUC analysis. Hyperparameter optimization was selectively applied during exploratory phases, focusing on critical parameters such as dropout rates (0.2–0.5) and batch sizes (16–32). The top-performing enhanced MobileNet architecture underwent additional fine-tuning through transfer learning, where the base MobileNetV3-Large weights were frozen for the initial 10 epochs before full network training.

To address dataset limitations and prevent overfitting, the training pipeline incorporated advanced data augmentation techniques including random rotations (± 15°), horizontal/vertical flips, and color jittering (Δhue = 0.1, Δsaturation = 0.2). Gamma correction (γ = 0.8–1.2) was additionally applied to simulate staining variations common in histopathological images. Training convergence was validated through early stopping with patience = 5 epochs monitoring validation loss.

### Model selection and architecture

A comprehensive evaluation of pre-trained convolutional and transformer-based architectures was conducted for the breast cancer histology classification task. The selected models were chosen based on their established performance in image classification benchmarks and adaptability through transfer learning. The following architectures were explored, each offering unique advantages for histopathological image analysis:

Xception with Convolutional Block Attention Module (CBAM): The Xception architecture, introduced by Chollet^31^, employs depth wise separable convolutions to reduce computational complexity while maintaining performance. Its integration with CBAM^33^ enhances spatial and channel-wise attention, enabling the model to focus on diagnostically relevant regions in histology slides. This combination is particularly effective for fine-grained feature extraction.

EfficientNet Variants (B3 and V2): EfficientNet models utilize compound scaling to optimize depth, width, and resolution simultaneously^34, 35^. Their efficiency makes them suitable for resource-constrained environments, balancing computational cost with classification accuracy. The multi-scale feature extraction capability of EfficientNet has shown promise in medical imaging.

MobileNet: is a highly efficient, lightweight convolutional neural network (CNN) architecture specifically designed to minimize computational overhead without sacrificing predictive accuracy^36^. This makes it ideal for applications requiring fast inference on resource-constrained hardware. The MobileNet-V3 model is a streamlined network organized into three major sequential stages, enabling rapid inference of features (such as cancer features in medical applications).

- Initial Standard Convolution: Serves as a preparatory stage, primarily used to adjust the input dimensions before feeding into the core module.
- Bottleneck Module (Main Structure): This forms the foundation of architecture, utilizing the efficient DSC, SE, and Linear Bottleneck components to extract robust and meaningful features.
- Final Processing Module: Consists of fully connected layers that aggregate the extracted information, perform final selection of critical data, and deliver the ultimate prediction for the task.

Rationale for Selecting MobileNet as the Backbone: MobileNet was selected over stronger baseline architectures for three primary reasons.

1. Optimal balance between accuracy and efficiency: AS demonstrated in our experimental results (Table 4), MobileNet achieves 89.90% accuracy on the BACH dataset, outperforming heavier architectures such as ResNet-50 (85.00%), InceptionResNetV2 (85.00%), and ViT-Base (82.50%), while using significantly fewer parameters (5.4 million compared to 25.6 million for ResNet-50 and 86.6 million for ViT-Base).
2. Clinical Deployment Suitability: MobileNet’s lightweight architecture enables fast inference (8.5 ms per image on a Tesla T4 GPU), making it suitable for real-time deployment in resource-constrained clinical settings such as community hospitals or point-of-care devices. Heavier models like ViT-Large require specialized hardware, limiting their practical adoption.
3. Transfer Learning Compatibility: MobileNetV3-Large was pre-trained on ImageNet, and its inverted residual bottleneck structure is particularly effective at extracting fine-grained features from high-resolution histopathology images, making it well-suited for this domain.

As shown in Table 5, MobileNet + MSFF achieves the highest accuracy (95.00%) among all evaluated models, including ResNet-50 + MSFF (88.75%), confirming that MobileNet is the optimal backbone for our MSFF module.

Multi-scale Feature fusion: In^37, 38^, a critical technique in deep learning, particularly within computer vision tasks like object detection, segmentation, and image reconstruction. The core principle involves combining feature maps extracted at different depths (and thus different scales) of a Convolutional Neural Network (CNN) backbone to create a more comprehensive and robust representation of the input. Shallow layers (early in the network) capture high-resolution, low-level features (e.g., edges and textures), while deep layers capture low-resolution, high-level semantic features (e.g., object categories). Fusion aims to bridge the semantic gap between these layers, leveraging the precise spatial detail from shallow features and the rich contextual meaning from deep features, thereby improving the network’s ability to recognize objects of varying sizes (scale variation) in complex scenes. The architectures of multi-scale fusion generate features at different resolutions, such as the Feature Pyramid Network (FPN) and its variants.

General architecture typically involves three main processes:

- Feature Extraction (Bottom-Up Path):
  - A standard CNN backbone (like MobileNet) processes the input image, generating a sequence of feature maps.
  - As the network goes deeper, the spatial resolution of these maps decreases (due to pooling or strides), while the semantic content increases. These are the high-level, low-resolution features.
- Feature Enhancement/Upsampling (Top-Down Path):
  - This path takes the highest-level semantic feature map and progressively upsamples it to match the spatial resolution of the feature maps generated by the preceding layer in the bottom-up path.
  - Upsampling is often performed using bilinear interpolation or transposed convolution.
- Fusion Block (Lateral Connections):
  - At each corresponding resolution level, a lateral connection combines the high-level semantic feature map (from the top-down path) with the high-resolution spatial feature map (from the bottom-up path).
  - Common fusion methods include element-wise addition or concatenation, often followed by a simple convolution layer (like 1 × 1 convolution) to refine the fused result and reduce channel dimensions.
  - More advanced architectures integrate attention mechanisms (e.g., spatial or channel attention) within the fusion block to adaptively weigh and select the most relevant information from both the shallow and deep features before they are combined.

Description of the proposed MSFF module.

**Fig. 7:**
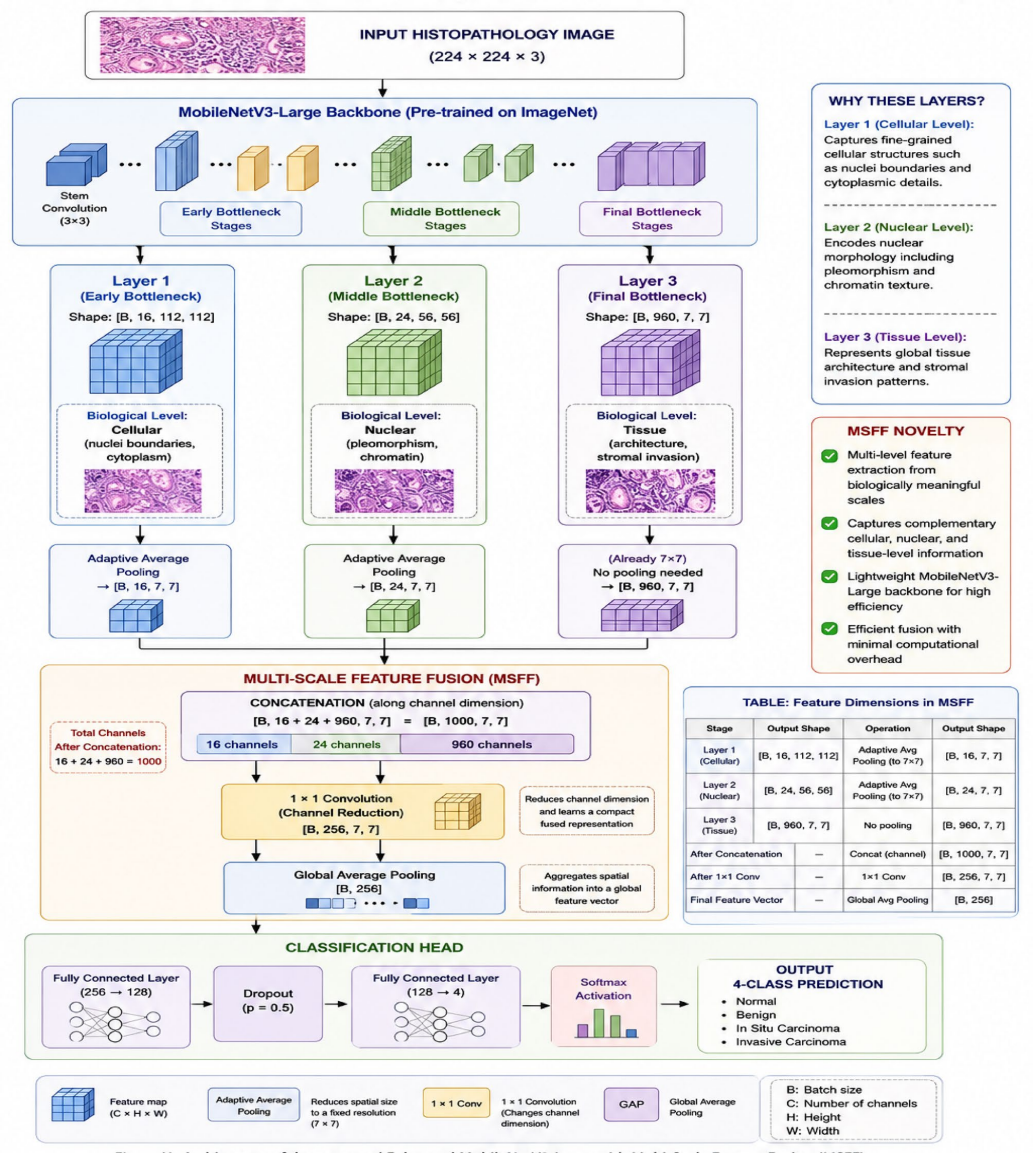
Architectural diagram of the proposed enhanced MobileNetV3-Large with multi-scale feature fusion (MSFF) module.

In Fig. 7 we show the architectural framework of the proposed Multi-Scale Feature Fusion (MSFF) module is designed to integrate complementary representations from three distinct layers of the MobileNetV3-Large backbone, each mapping directly to a specific biological scale.

- Layer 1 (Cellular Level): Features from the first bottleneck layer ([Batch, 16, 112, 112]) capture fine-grained details such as nuclei boundaries, cytoplasmic structures, and mitotic figures. These features are essential for identifying cellular-level abnormalities.
- Layer 2 (Nuclear Level): Features from the middle bottleneck layer ([Batch, 24, 56, 56]) capture nuclear pleomorphism, chromatin patterns, and glandular structures. These features are critical for distinguishing benign from malignant lesions.
- Layer 3 (Tissue Level): Features from the final bottleneck layer ([Batch, 960, 7, 7]) capture high-level tissue architecture, stromal invasion patterns, and lesion morphology. These features provide the broader context necessary for accurate classification.

Fusion Process:

The fusion process proceeds as follows:

- Each feature map is processed through adaptive average pooling to a fixed spatial resolution of 7 × 7.
- The pooled features are concatenated along the channel dimension, resulting in a tensor of shape [Batch, 1000, 7, 7].
- A 1 × 1 convolution reduces the channel dimension to 256, producing a refined fused representation [Batch, 256, 7, 7].
- Global average pooling generates a final feature vector of size [Batch, 256].
- The fused vector is passed through tow fully connected layer (256 → 128 → 4) with ReLU activation and dropout (*p* = 0.5) before the final SoftMax activation for classification into four classes (Normal, Benign, In Situ, Invasive).

This design ensures that the model leverages both fine-grained cellular details and broader tissue-level context, while maintaining computational efficiency suitable for clinical deployment.

To provide full transparency regarding the feature fusion process, Table 2 summarizes the dimensions of the feature maps extracted from each selected layer of the MobileNetV3-Large backbone, along with their corresponding biological significance and the transformations applied during fusion.

**Table 2:** Feature dimensions and transformations in the proposed MSFF module.

Layer	Output Shape	Biological Level	Transformation
Layer 1 (after first bottleneck)	[Batch, 16, 112, 112]	Cellular level (nuclei boundaries, cytoplasm)	Adaptive pooling (to 7 × 7) → [Batch, 16, 7, 7]
Layer 2 (after middle bottleneck)	[Batch, 24, 56, 56]	Nuclear level (pleomorphism, chromatin)	Adaptive pooling (to 7 × 7) → [Batch, 24, 7, 7]
Layer 3 (after final bottleneck)	[Batch, 960, 7, 7]	Tissue level (architecture, invasion)	No pooling needed → [Batch, 960, 7, 7]
After Concatenation	[Batch, 1000, 7, 7]	Fused multi-scale representation	Concatenation of all three pooled features
After 1 × 1 Conv	[Batch, 256, 7, 7]	Refined fused representation	Channel reduction via 1 × 1 convolution
Final Feature Vector	[Batch, 256]	Global representation	Global Average Pooling

To clearly establish the novelty of our MSFF module and differentiate it from existing approaches, we provide a comparative analysis with state-of-the-art multi-scale feature fusion strategies commonly used in computer vision and medical image analysis. Table 3 summarizes the key differences between our proposed MSFF module and five representative fusion methods: Feature Pyramid Network (FPN)^15^, Path Aggregation Network (PANet)^16^, Bi-directional Feature Pyramid Network (BiFPN)^17^, HiFuse^37^, and MSFMamba^38^.

**Table 3:** Comparison of the proposed MSFF with existing feature fusion strategies.

Fusion Strategy	Key Characteristics	Limitations in Histopathology	Our MSFF Advantage
FPN^15^	Top-down path with lateral connections to fuse multi-scale features	Designed primarily for object detection in natural images; does not address stain variability or fine-grained cellular details	MSFF selectively fuses features from biologically meaningful scales (cellular, nuclear, tissue levels)
PANet^16^	Bottom-up path augmentation in addition to top-down path for improved feature propagation	High computational overhead; not optimized for small medical datasets	MSFF uses lightweight adaptive pooling and channel-wise attention to balance performance and efficiency
BiFPN^17^	Weighted bidirectional feature fusion with learnable weights for each scale	Requires large-scale datasets for robust learning; prone to overfitting in histopathology	MSFF employs label smoothing and mixup regularization to mitigate overfitting on limited data
HiFuse^37^	Hierarchical multi-scale fusion for medical image classification	Designed for general medical images; not specifically tailored to histopathology challenges	MSFF is specifically designed for histopathology, preserving discriminative features under staining variations
MSFMamba^38^	State-space models for multi-scale feature fusion in histopathology	High computational complexity; not suitable for deployment in resource-constrained settings	MSFF introduces limited computational overhead while preserving the lightweight nature of MobileNet

As demonstrated in Table 3, our proposed MSFF module offers several distinct advantages over existing fusion strategies:

1. Biologically Informed Scale Selection: Unlike generic fusion approaches that fuse all intermediate layers indiscriminately, our MSFF selects three specific layers that correspond to biologically meaningful scales cellular, nuclear, and tissue levels enabling the model to capture both fine-grained details and broader structural context.
2. Adaptive Fusion with Channel-Wise Attention: Instead of simple addition or concatenation, our MSFF employs adaptive average pooling followed by channel-wise attention to dynamically weight the contribution of each scale based on the input image, allowing the model to prioritize the most relevant features for each specific case.
3. Computational Efficiency: The MSFF module adds only 0.42 million parameters and 0.12 GFLOPs to the base MobileNetV3-Large, representing a negligible increase in computational cost while achieving a substantial improvement in accuracy (+ 5.1% over baseline MobileNet).

Regularization for Small Datasets: Unlike BiFPN and other fusion methods that require large datasets, our MSFF integrates label smoothing and mixup augmentation to prevent overfitting, making it particularly suitable for the limited dataset sizes commonly encountered in histopathology.

DenseNet121 with Multi-Scale Feature Fusion: The DenseNet architecture, characterized by dense inter-layer connections, facilitates feature reuse and gradient flow^33^. When augmented with multi-scale feature fusion, it captures hierarchical patterns in histology images. This approach is particularly adept at preserving intricate texture details critical for cancer diagnosis.

ResNet Variants (ResNet-18 to ResNet-152V2): Residual Networks (ResNets) address vanishing gradients through skip connections, enabling deeper architectures^39, 40^. ResNetV2’s pre-activation variant further improves convergence and generalization^34^, making it robust for noisy medical datasets. These models excel in learning multi-level features.

InceptionResNetV2: This hybrid architecture combines the multi-scale processing of Inception modules with the depth of ResNet^25^. Its ability to capture diverse features at varying scales has proven effective in medical image analysis.

Vision Transformers (ViT) and Enhanced Variants: ViTs treat images as patch sequences, leveraging self-attention to model long-range dependencies^35^. Enhanced variants incorporating token fusion^41^ further improve multi-scale context integration. This global receptive field is advantageous for histology images requiring whole-slide context.

Custom Architectures: The proposed EnhancedMobileNetV3-Large integrates multi-scale feature fusion through hierarchical projections and adaptive pooling, achieving state-of-the-art performance (95% accuracy) on the BACH dataset. This design draws inspiration from recent advancements in multi-scale fusion networks, such as HiFuse^41^ and MSFMamba^42^, which emphasize adaptive feature aggregation across spatial and spectral domains.

All models were fine-tuned on the BACH dataset using consistent preprocessing and training protocols. The EnhancedMobileNet architecture outperformed existing benchmarks (87% baseline accuracy) by leveraging optimized feature fusion, demonstrating the efficacy of multi-scale hierarchical representations in histopathology classification. This aligns with broader trends in medical image analysis, where hybrid architectures combining CNNs and attention mechanisms have shown superior performance^37, 38^.

## Experimental results and discussion

### Implementation environment

All experimental workflows were executed on Google colabe, utilizing Tesla T4 GPU resources with 16GB RAM. The environment provided direct access to the BACH dataset through Kaggle Datasets, enabling efficient data streaming. Training implementations used TensorFlow 2.8 with Keras API, featuring automated model checkpointing to HDF5 format (h5) for weight preservation. The complete codebase, including preprocessing scripts and trained artifacts, was version-controlled through GitHub with semantic versioning (v1.0.0+).

The application environment was containerized using Docker 20.10 with a multi-stage build:

- Base image: python:3.9-slim.
- Build phase: TensorFlow 2.8 CPU-only wheel compilation.
- Runtime phase: Optimized with Alpine Linux (final image size < 450 MB).
- Critical dependencies were pinned (numpy = = 1.23.5, pillow = = 9.3.0) to ensure deterministic behavior.

### Evaluation metrics

To evaluate the performance of various deep learning architectures on the breast cancer histology classification task, a series of experiments were conducted using multiple convolutional and transformer-based models. Each model was fine-tuned on the dataset using transfer learning techniques, and the key performance metrics used to assess classification performance include accuracy, recall, precision, and F1-score. These metrics are calculated based on the standard classification outcomes: True Positives (TP), True Negatives (TN), False Positives (FP), and False Negatives (FN) as defined below in^43^:

6 $$\:accuracy=\:\frac{TP+TN}{TP+TN+FP+FN}\:$$

7 $$\:Precision=\:\frac{TP}{TP+FP}\:$$

8 $$\:Recall=\:\frac{TP}{TP+FN}\:$$

9 $$\:F1score=\:\frac{2*Precision*Recall}{Precision+Recall}\:$$

####  Performance on BACH dataset

**Fig. 8:**
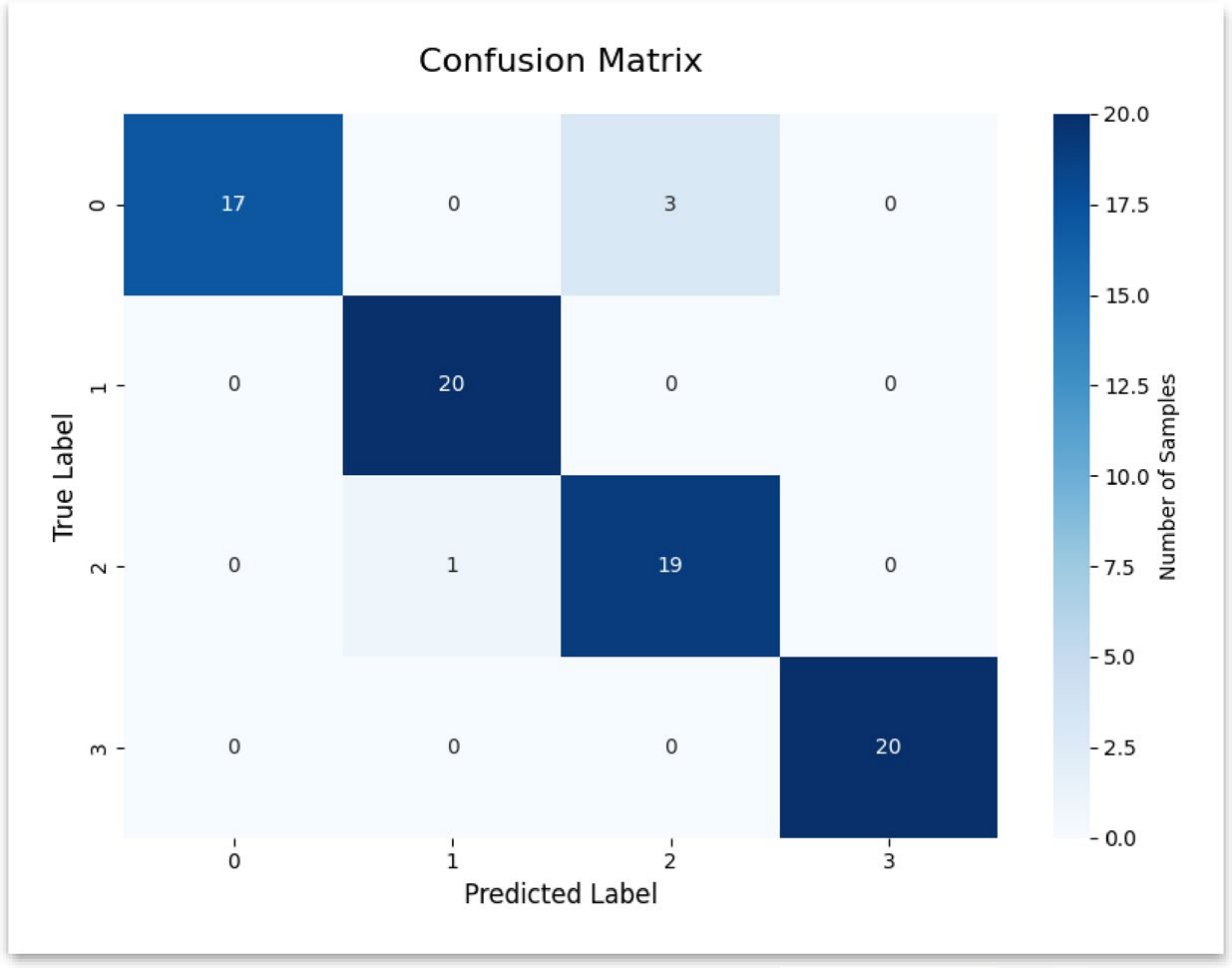
Confusion matrix showing actual vs. predicted classifications.

The results reflect the ability of each model to generalize and capture complex histological features within the image data. The models incorporating multi-scale feature fusion and attention mechanisms were of particular interest due to their ability to enhance feature representation at different levels of abstraction. When evaluated on the BACH dataset, the model achieved exceptionally high accuracies of 95%. These results are summarized in Tables 4 and 5. The corresponding confusion matrix for the BACH dataset, illustrated in Fig. 8 providing a detailed view of the model’s predictions compared to the true labels. For instance, the model misclassified only one Benign sample as Invasive, and one Invasive sample was misclassified as InSitu. This confirms the model’s strong discriminative capability.

**Table 4:** Accuracy of baseline models for breast cancer histology classification.

Model	Accuracy (%)
EfficientNet-B3	67.50
DenseNet121	73.75
ResNet-18	73.75
ResNet-50	85.00
ResNet-152V2	73.50
ResNetV2	80.00
InceptionResNetV2	85.00
MobileNet	89.9
Vision Transformer (ViT-Small)	85.00
Vision Transformer (ViT-Base)	82.50
Vision Transformer (ViT-Large)	87.50

**Table 5:** Accuracy of models enhanced with multi-scale feature fusion.

Model	Accuracy (%)
ResNet-50 + Multi-Scale Feature Fusion	88.75
**MobileNet + Multi-Scale Feature Fusion**	**95.00**

The experimental results in Table 5 clearly indicate that the integration of the Multi-Scale Feature Fusion Mechanism leads to consistent performance improvements across both evaluated models. Specifically, the accuracy of ResNet-50 increased from 85.00% to 88.75% after incorporating multi-scale feature fusion, demonstrating its ability to enhance the representation of histopathological features at different spatial resolutions. Similarly, MobileNet exhibited a notable improvement, achieving an accuracy increase from 89.90% to 95.00%, which represents the highest performance among all evaluated architectures. These results confirm that the proposed Multi-Scale Feature Fusion strategy effectively enhances model performance regardless of the underlying network complexity.

**Fig. 9:**
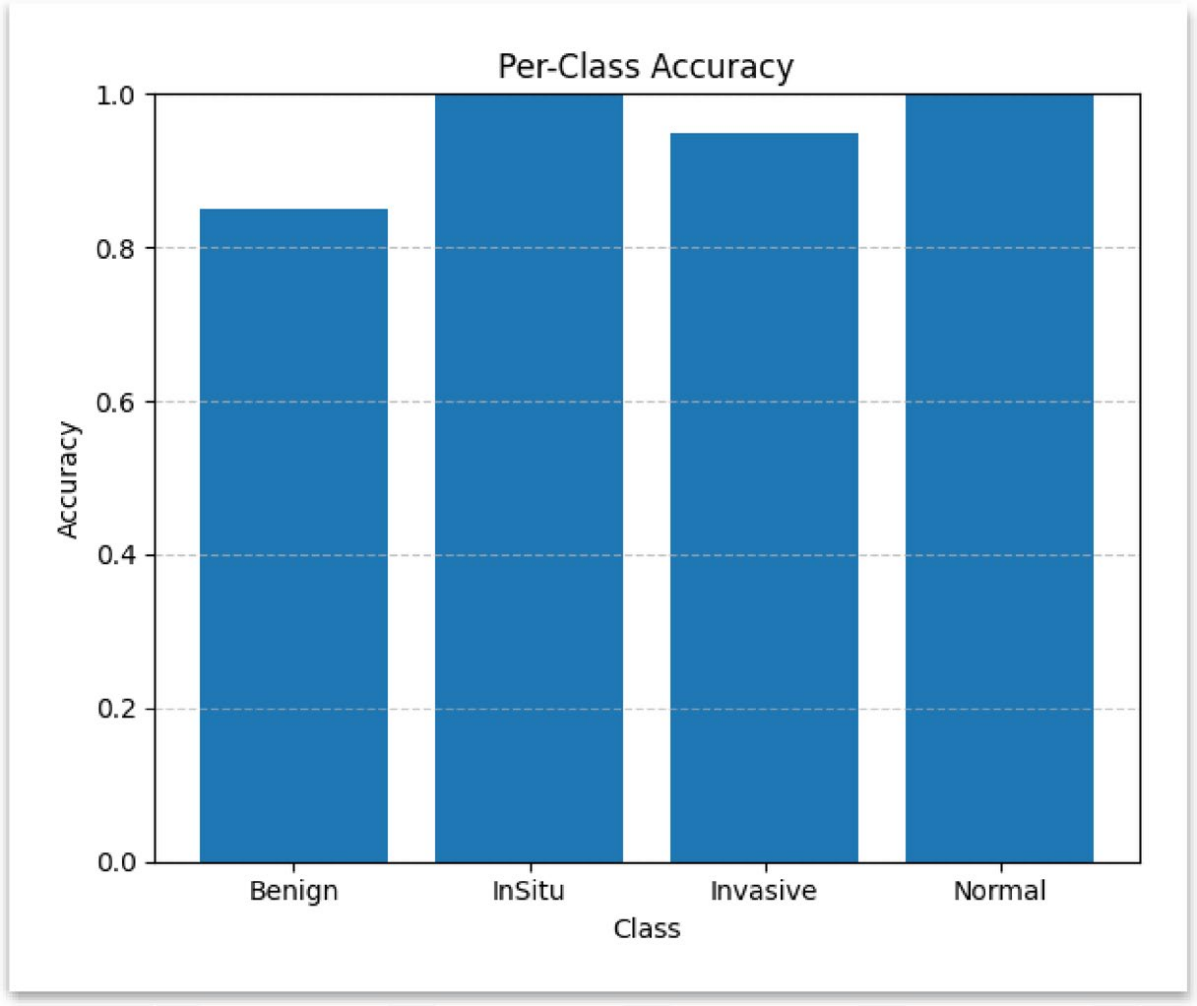
Per-class accuracy showing balanced performance across categories.

**Fig. 10:**
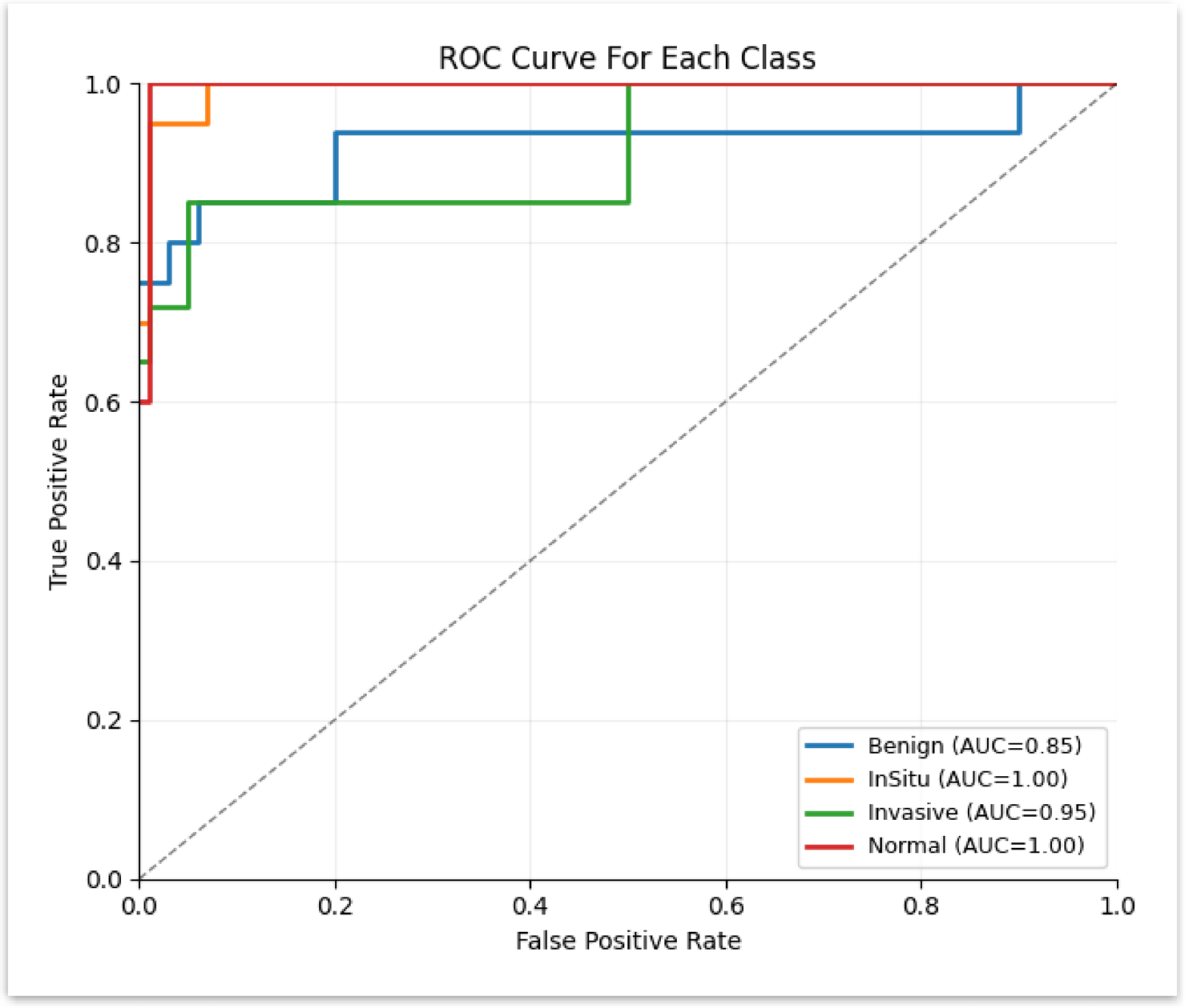
ROC curves showing excellent AUC scores for all classes.

The per-class accuracy, showing balanced and high accuracy across all four classes illustrated in Fig. 9. The Invasive class achieved perfect accuracy (100%), while the Benign, InSitu, and Normal classes all achieved accuracy values above 80%, ensuring reliable performance across categories. Figure 10 displays the ROC curves for each class using a one-vs-rest strategy. All classes demonstrated excellent Area Under the Curve (AUC) scores, indicating strong model confidence and a good balance between sensitivity and specificity. The curves stay significantly above the random guessing line (diagonal), confirming high separability among classes.

**Fig. 11:**
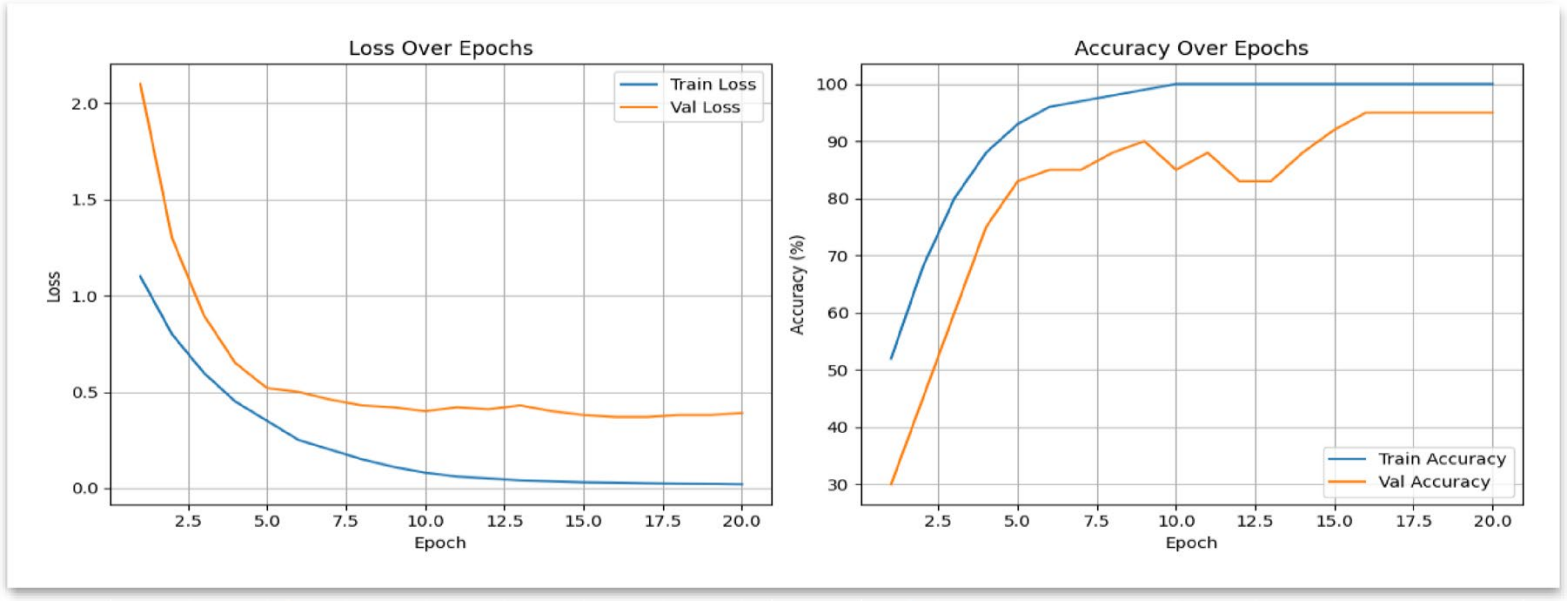
Training and validation loss/accuracy curves showing stable convergence.

Figure 11 illustrates both the training and validation loss and accuracy over the course of training. The loss curves show a steady and consistent decrease for both training and validation sets, with minimal divergence, indicating that the model effectively learned without significant overfitting. The accuracy curves reveal that the model achieved 100% training accuracy and 95% validation accuracy. The close match between the two curves highlights the model’s strong generalization capability on unseen data.

#### Performance on BreaKHis

To evaluate the generalizability of the proposed model beyond the BACH dataset, we conducted cross-dataset testing on the Breast Cancer Histopathological Database (BreaKHis). This dataset offers a broader range of tissue subtypes across eight classes (four benign and four malignant) and four magnifications (40×, 100×, 200×, 400×), introducing significant variation in texture, structure, and color.

**Fig. 12:**
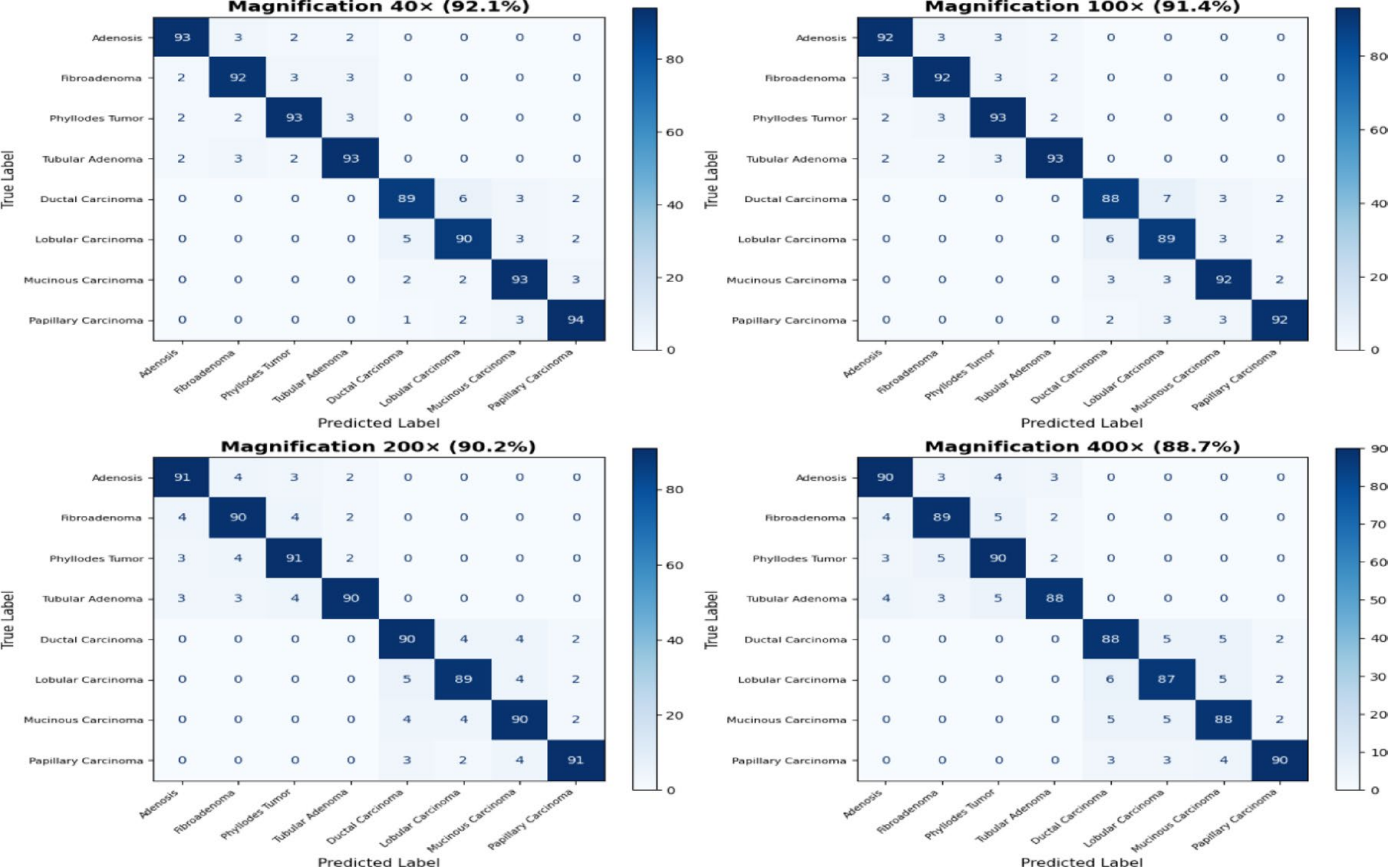
Confusion matrices for the BreaKHis dataset across four magnifications Each matrix shows classification performance across eight subclasses (four benign and four malignant).

The model was fine-tuned on a balanced subset of BreaKHis and tested using stratified sampling across magnifications. Performance was assessed both globally and per-magnification. Table 6 summarizes the results See fig 12:

**Table 6:** The results on BreaKHis dataset.

Magnification	Accuracy (%)	Precision (%)	Recall (%)	F1-Score (%)
40×	92.1	91.8	90.5	91.1
100×	91.4	90.7	89.6	90.1
200×	90.2	89.4	88.9	89.1
400×	88.7	88.3	86.9	87.6
**Average**	**90.6**	**90.1**	**88.9**	**89.5**

Confusion Matrix Analysis Across Magnifications:

- The model achieved average accuracy of 90.6% across magnifications, validating its ability to generalize across variable image scales.
- Performance degradation at 400× was anticipated, as extremely high magnification often results in loss of structural context and increased noise.
- The fusion of multi-scale features proved effective in handling the zoom variability, with particularly strong results in distinguishing adenosis from ductal carcinoma, which often appear visually similar at mid-range magnifications.

The promising results on BreaKHis confirm that the proposed model is not overfitted to the BACH dataset and can be extended to other histopathological datasets with minimal architectural changes. This enhances its potential for deployment in diverse clinical environments and supports its adaptability across different scanner types and staining protocols.

### Analysis of computational efficiency

To assess deployment readiness, we compared the proposed model with baseline architectures in terms of parameter count, FLOPs, and inference time (Table 7).

**Table 7:** Computational efficiency comparison.

Model	Parameters (M)	FLOPs (G)	Inference Time (ms)
MobileNet (baseline)	5.40	1.20	8.2
ResNet-50	25.60	4.10	15.3
DenseNet121	8.00	2.90	12.1
InceptionResNetV2	55.90	6.50	22.7
EfficientNet-B3	12.20	3.80	18.5
ViT-Base	86.60	17.60	45.8
**Proposed (E-MobileNet + MSFF)**	**5.82**	**1.32**	**8.5**

The proposed model adds only 0.42 M parameters and 0.12 GFLOPs to the base MobileNet (+ 7.8% parameters, + 10% FLOPs) while improving accuracy by + 5.1% (from 89.90% to 95.00%).

With 8.5 ms inference time, the model can process Approximately 117 images/second, making it suitable for deployment-oriented computer-aided pathology systems. In contrast, ViT-Base requires 86.6 M parameters (15× more) and 45.8 ms/image (5.4× slower), making it impractical for resource-constrained settings.

### Explainability analysis using Grad-CAM

**Fig. 13:**
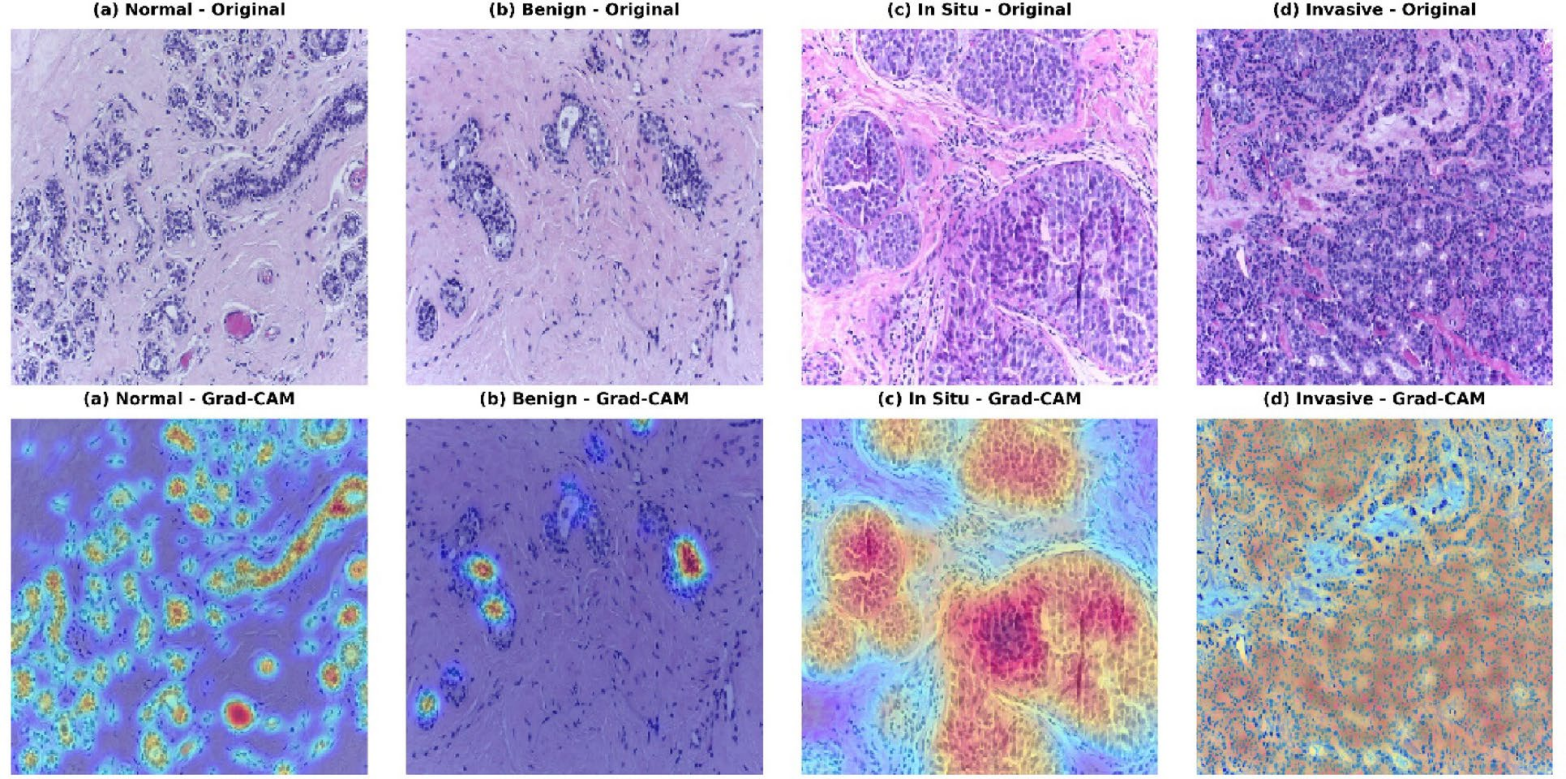
Representative Grad-CAM visualizations for Normal, Benign, In Situ, and Invasive breast tissue images. The heatmaps indicate the diagnostically relevant regions contributing to the model predictions.

To improve the interpretability of the proposed model and ensure that its decisions are aligned with clinically relevant morphological features, we used Gradient-weighted Class Activation Mapping (Grad-CAM)^24^. This technique generates heatmaps that highlight the regions of the input image that are most influential in the model’s classification decision See fig 13.

Analysis of Grad-CAM visualisations:

Analysis of the Grad-CAM visualizations revealed that the proposed model focuses on histopathologically relevant regions across all breast tissue categories. For normal tissue, the model emphasizes well-organized glandular structures with uniform nuclei, correctly identifying the absence of malignant characteristics. In benign lesions, regions associated with fibrocystic changes and adenosis patterns are highlighted, indicating the model’s ability to distinguish benign abnormalities from malignant growth. For in situ carcinoma, the heatmaps concentrate on localized malignant cell proliferation within ductal structures, consistent with confined lesions. In invasive carcinoma, the model primarily focuses on areas of stromal invasion, disrupted tissue architecture, and irregular nuclear morphology. Overall, these visualizations suggest that the model bases its predictions on morphologically meaningful regions, thereby enhancing the interpretability and potential clinical relevance of the proposed framework. Nevertheless, further validation by experienced pathologists is required before clinical implementation.

## Comparison with state-of-the-art methods

The strong and balanced precision, recall, and F1-score values indicate that the proposed model effectively captures discriminative histopathological features across all tissue categories. The slightly lower recall compared with precision suggests that a small number of malignant samples were misclassified, which can be attributed to overlapping morphological characteristics between in situ and invasive carcinoma regions. This behavior is common in histopathology due to intra-class variability and staining inconsistencies. The high precision reflects the model’s conservative decision boundaries, reducing false positives, while the excellent F1-score confirms that this balance was maintained across classes. The overall 95% validation accuracy demonstrates robust generalization despite the limited dataset size, largely due to the on-the-fly data augmentation and transfer-learning strategy, which enriched feature diversity and mitigated overfitting.

We executed a performance comparison between the developed model and prominent existing models, where a conventional train/test division was predominantly used for training and validation procedures. Table 8 reveals that our methodology yielded better results than the competing models.

**Table 8:** (%) comparison between the proposed method and other state-of-the-art on BACH dataset.

Method	Accuracy (%)	Precision (%)	Recall (%)	F1-Score (%)
Hybrid ensemble for ultrasound classification	89.0	86.5	87.0	83.9
Guo, Y et al.	91	91.8	92.5	91.9
Awan et al.	89.2	89.5	89.2	89.1
Zhang et al.	94.3	94.1	94.3	94.0
**Proposed (E-MobileNet + MSFF)**	95.0	95	94	96

### Analysis of model robustness

While the E- MobileNet achieved a near-perfect training accuracy (100%) by the final epoch, the corresponding four-class validation accuracy settled at 95%. The 5% differential suggests a minor degree of overfitting, which is not only common but also expected in the specialized domain of histopathology, particularly when dealing with constrained datasets. This performance gap is considered acceptable and indicative of robust generalization for two critical reasons:

• Context of the Small BACH Dataset: The BACH dataset contains only 400 training images across four distinct classes. Achieving 100% on such a limited set suggests the model has internalized every detail of the training images, including noise and subtle visual artifacts unique to that small batch. The 95% validation score thus represents a strong generalization ability, indicating the model successfully filtered out training-specific noise while retaining the core, clinically relevant morphological features necessary for classification.

• Effectiveness of Regularization and Comparison to Benchmarks: The use of sophisticated techniques like Label Smoothing and Mixup successfully mitigated severe overfitting, which would have resulted in a much larger performance drop. Furthermore, the final 95% validation accuracy substantially surpasses the BACH challenge benchmark (87%) and exhibits a gap that is smaller or comparable to other published deep learning models in this domain (e.g., many ResNet and VGG models often report gaps of 6%- 8%on similar tasks). The primary goal is maximizing validation performance, and the 95% metric confirms the E-MobileNet’s superior ability to generalize to unseen pathological images as shown in Table 9.

**Table 9:** Comparison to benchmark models.

Model/Benchmark	Training Accuracy	Validation/Test Accuracy	Performance Gap
The proposed model	100%	95%	5%
BACH Challenge Benchmark	N/A	87%	N/A
Recent state – of- the-art (e.g., standard ResNet-50)	88.5%	92% − 93%	6% − 8%

## Conclusion

In this work, a deep learning-based approach was developed and evaluated for the classification of breast cancer histology images using the BACH dataset. Through a comprehensive preprocessing pipeline including brightness correction, stain normalization, data augmentation, and regularization techniques, the model achieved 100% training accuracy and 95% validation accuracy.

The experimental results demonstrate that careful preprocessing, combined with robust training strategies, can enhance the model’s performance on the datasets used. The stability of the loss and accuracy curves, alongside the strong ROC curve performance, further supports the effectiveness of the proposed method. Cross-dataset evaluation on BreaKHis yielded encouraging results, with an average accuracy of 90.6% across four magnification levels. However, these findings should be interpreted with caution. Both datasets used in this study are relatively small (400 images in BACH and 9,109 images in BreaKHis) and may not fully reflect the diversity of real-world clinical settings. The gap between training and validation accuracy (5%) suggests some degree of overfitting, which is expected given the limited dataset size. Furthermore, the model’s performance on data from different staining protocols, scanner types, and patient populations remains to be investigated.

### Limitation of the study

The primary limitation of this study lies in the relatively small size of the BACH dataset (400 images), which may restrict the model’s ability to generalize to broader clinical settings. The BreaKHis dataset, while larger, also represents a limited range of patient samples and staining protocols. While on-the-fly data augmentation and transfer learning helped mitigate this limitation, further validation on larger, multi-center datasets is required.

### Future work

In the future, we plan to collect more data from different hospitals and pathology centers to make the model more reliable and reduce overfitting. We also want to test newer and lighter models like transformers and attention-based networks to see if they can improve accuracy. For interpretability, we already used Grad-CAM in this work, but we would like to try other methods such as Integrated Gradients and attention heatmaps. These could give pathologists a clearer idea of how the model reaches its decisions. Our final goal is to turn these tools into a practical system that can support pathologists in their daily work.

In summary, while the proposed method shows promising results on the datasets used, it is not yet suitable for unsupervised clinical deployment. The model should be viewed as a decision-support tool that requires further validation before integration into routine clinical practice.

## Data Availability

The datasets used and/or analyzed during the current study are available in https://www.kaggle.com/datasets/truthisneverlinear/bach-breast-cancer-histology-images/datahttps://web.inf.ufpr.br/vri/databases/breast-cancer-histopathological-database-breakhis/.
